# The Specificities of Lysophosphatidic Acid Acyltransferase and Fatty Acid Desaturase Determine the High Content of Myristic and Myristoleic Acids in *Cyanobacterium* sp. IPPAS B-1200

**DOI:** 10.3390/ijms25020774

**Published:** 2024-01-07

**Authors:** Alexander Y. Starikov, Roman A. Sidorov, Kirill S. Mironov, Dmitry A. Los

**Affiliations:** K.A. Timiryazev Institute of Plant Physiology, Russian Academy of Sciences, Botanicheskaya Street 25, 127276 Moscow, Russia; starikovay1393@gmail.com (A.Y.S.); roman.sidorov@mail.ru (R.A.S.); ksmironov@gmail.com (K.S.M.)

**Keywords:** *Cyanobacterium*, *Synechococcus elongatus*, acyltransferase, fatty acids, fatty acid desaturase, myristic acid, myristoleic acid

## Abstract

The cyanobacterial strain *Cyanobacterium* sp. IPPAS B-1200 isolated from Lake Balkhash is characterized by high relative amounts of myristic (30%) and myristoleic (10%) acids. The remaining fatty acids (FAs) are represented mainly by palmitic (20%) and palmitoleic (40%) acids. We expressed the genes for lysophosphatidic acid acyltransferase (LPAAT; EC 2.3.1.51) and Δ9 fatty acid desaturase (FAD; EC 1.14.19.1) from *Cyanobacterium* sp. IPPAS B-1200 in *Synechococcus elongatus* PCC 7942, which synthesizes myristic and myristoleic acids at the level of 0.5–1% and produces mainly palmitic (~60%) and palmitoleic (35%) acids. *S. elongatus* cells that expressed foreign LPAAT synthesized myristic acid at 26%, but did not produce myristoleic acid, suggesting that Δ9-FAD of *S. elongatus* cannot desaturate FAs with chain lengths less than C16. *Synechococcus* cells that co-expressed LPAAT and Δ9-FAD of *Cyanobacterium* synthesized up to 45% palmitoleic and 9% myristoleic acid, suggesting that Δ9-FAD of *Cyanobacterium* is capable of desaturating saturated acyl chains of any length.

## 1. Introduction

The biosynthesis of glycerolipids in cyanobacteria has been well studied [1,2]. The first reaction in it is the acylation of glycerol-3-phosphate at the *sn*-1 position with the formation of a molecule of lysophosphatidic acid. As a result of the subsequent acylation of the *sn*-2 position, phosphatidic acid is formed, which is a precursor of diacylglycerides. Thus, the substrate specificity of the acyltransferase may play an important role in the formation of the final FA composition of cyanobacteria.

Analysis of FAs from the cyanobacterium *Cyanothece* sp. PCC 8801 (more recent species name—*Rippkaea orientalis* PCC 8801) revealed that this species contained high levels of myristic acid (14:0; nearly 50% of total FAs) and linoleic acid in its glycerolipids, with minor contributions from palmitic acid (16:0), stearic acid, and oleic acid. Myristic acid was esterified primarily to the *sn*-2 position of the glycerol moiety of glycerolipids [3]. This characteristic is unique because, in cyanobacterial strains, the *sn*-2 position is usually occupied by C16 FAs, e.g., 16:0. Transformation of *Synechocystis* sp. PCC 6803 with the PCC8801_1274 gene for lysophosphatidic acid acyltransferase (LPAAT; 1-acyl-*sn*-glycerol-3-phosphate acyltransferase) from *Cyanothece* sp. PCC 8801 shifted the level of 14:0 from 1–2% to 17% in all lipid classes. These findings suggest that the high content of 14:0 in *Cyanothece* sp. PCC 8801 might be a result of the high specificity of this acyltransferase toward the 14:0-acyl-carrier protein [3].

Another representative of cyanobacteria with high C14 content is the prochlorophyte, *Prochlorothrix hollandica*, which contains 14:0 (10–14%), 14:1Δ9 (17–33%), 16:0 (23–30%), 16:1Δ9 (18–23%), 18:0 (1–10%), and 18:1 (3–6%) in membrane lipids depending on storage and growth conditions [4].

The representatives of *Cyanobacterium* spp. are characterized by the presence of only one gene for a fatty acid desaturase (FAD), namely, Δ9-FAD of type 1 [5,6,7,8], and display a simple FA profile: 14:0 (20%), 14:1Δ9 (10%), 16:0 (20%), 16:1Δ9 (45%), 18:0, and 18:1Δ9 (1–2% each) [5,8]. *Cyanobacterium* sp. IPPAS B-1200, a cyanobacterium isolated from samples obtained from the saline lake Balkhash, contains 30–40% C14 saturated myristic and monounsaturated myristoleic acids [5]. An even more simple FA profile is characteristic for the cyanobacterium *Synechococcus elongatus* PCC 7942—14:0 (<1%), 16:0 (45%), 16:1 (50%), 18:0 (1%), and 18:1Δ9 (3%) [9]. *S. elongatus* PCC 7942 has been widely used as a model to study the process of FA desaturation [9,10] and the properties of FADs [11,12].

Here we aimed to expressed LPAAT and Δ9-FAD from *Cyanobacterium* sp. IPPAS B-1200 (separately and jointly) in *Synechococcus elongatus* PCC 7942 in order to assess the suggestion that chain-length-specific LPAAT is involved in the synthesis of 14:0. With expression of Δ9-FAD from *Cyanobacterium* sp. in *Synechococcus elongatus*, we aimed to clarify the FA length specificity of FADs from different cyanobacterial species.

## 2. Results

### 2.1. Changes in FA Composition of Total Lipids in S. elongatus Transformants

**Figure 1:**
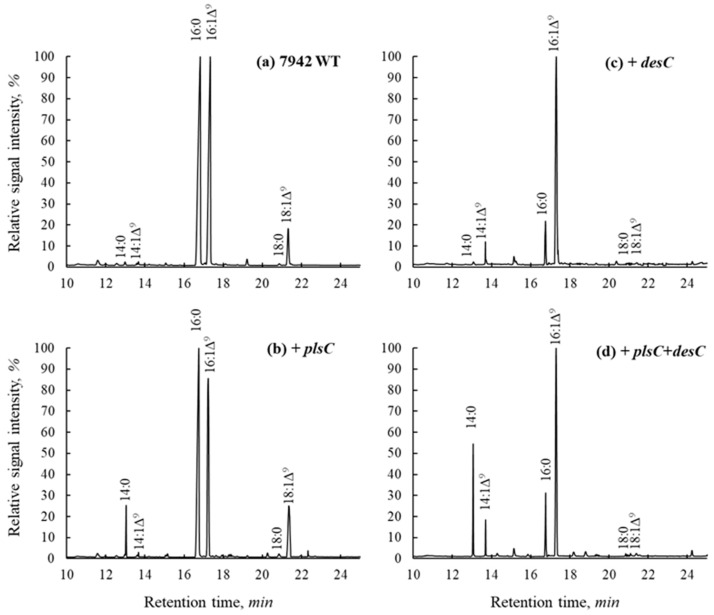
Separation of FA methyl esters obtained from the total lipids of *S. elongatus* wild-type (**a**) and transformant cells expressing *plsC* (**b**), *desC* (**c**), *plsC*, and *desC* (**d**) genes (total ion current).

The *plsC* and *desC* genes were cloned from the genome of the *Cyanobacterium* sp. strain IPPAS B-1200 enriched with myristic and myristoleic acids. These genes were successfully expressed in cells of the model cyanobacterium *S. elongatus* containing minor amounts of 14:0 and 14:1∆9 acids. A significant amount of myristic acid appeared when the gene for acyltransferase was used, and the balance shifted towards an increase in the proportion of monounsaturated acids in each of the transformant lines (Figure 1, Table 1).

*S. elongatus* transformed with the *plsC* gene for lysophosphotidyl-acyl-CoA:acyltransferase (LPPAT) displayed a nearly 20× increase in the amount of 14:0 compared with wild-type cells (from 0.5 to 10%). However, the absolute and relative amounts of 14:1 did not change much (from 0.5 to 0.8%), suggesting that Δ9-FAD of *S. elongatus* poorly uses 14:0 as a substrate.

*S. elongatus* transformed with the *desC* gene of *Cyanobacterium* for Δ9-FAD had its amount of 14:1 increased from 0.5 to 9%, indicating that this FAD successfully desaturated minor amounts of 14:0 synthesized by the wild-type cells of *Synechococcus*. Moreover, the amount of 16:1 increased from 32 to 73% (Table 1), suggesting that, in addition to myristic acid, Δ9-FAD of *Cyanobacterium* efficiently utilized palmitic acid as a substrate.

Co-expression of LPAAT and Δ9-FAD of *Cyanobacterium* in *S. elongatus* cells resulted in a significant increase in 14:0 (more than 26%) and 14:1 (9%). The level of 16:0 dropped from 50 to 15%, while the level of 16:1 increased from 32 (WT) to 49% (Figure 1, Table 1).

Apparently, these shifts in the C16/C14 proportions in *S. elongatus* were due to the appearance of significant amounts of C14 FAs, caused by the activities of LPAAT and Δ9-FAD of *Cyanobacterium* sp.

### 2.2. Stereospecific Positioning of C14 FAs

LPAAT is an enzyme that catalyzes the acylation of lysophosphatidic acid to the *sn*-2 position. In cyanobacteria, usually, this position is occupied by unsaturated 16:0 [13,14]. However, the PlsC1200 enzyme is presumably characterized by specificity for myristic acid. To confirm the specificity of the enzyme toward the *sn*-position, we isolated lysophosphatidic acid (LPA) and phosphatidic acid (PA) from *S. elongatus* wild-type cells and the transformant cell line expressing LPAAT.

**Figure 2:**
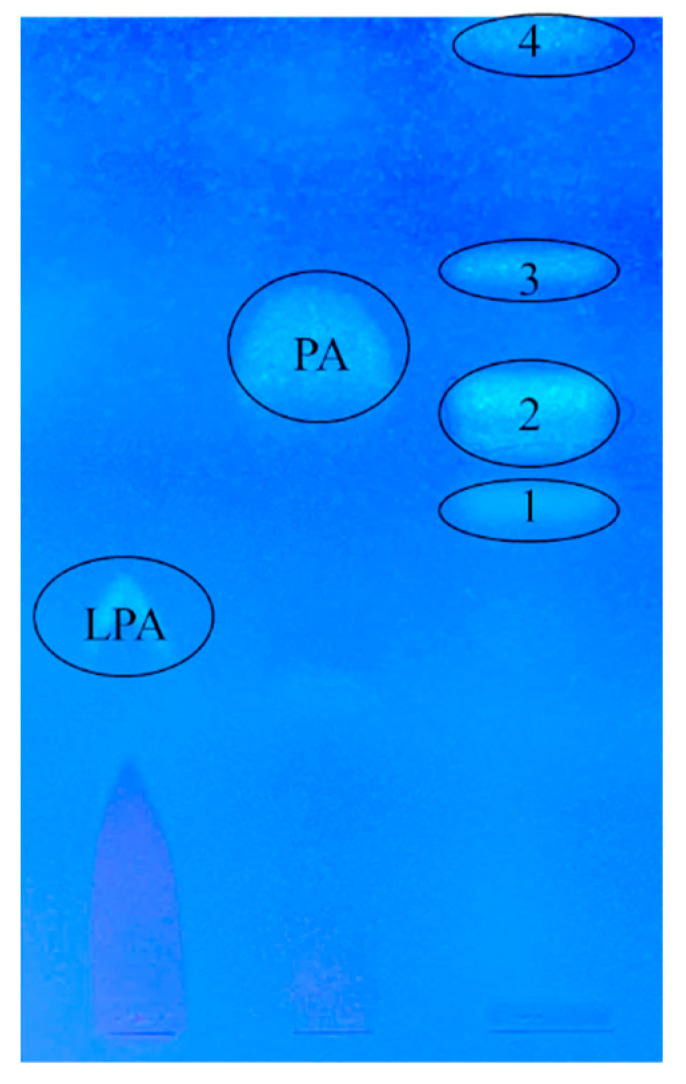
TLC separation of phospholipid fractions from cells of *S. elongatus* transformed with *plsC1200* (LPAAT) from *Cyanobacterium* sp. IPPAS 1200. The mobile phase is chloroform:methanol:acetone:water:acetic acid (6:2:8:1:1:1). Primulin-stained samples were illuminated at 366 nm. PA—phostphatidic acid standard, LPA—lysophosphatidic acid sodium salt standard. 1–4—isolated and analyzed phospholipid fractions. Marked areas have been withdrawn and analyzed.

Four distinct areas were detected after the separation of phospholipids, which differed in R_f_s with the standards PA and LPA (Figure 2). These mismatches may be due to the difference in acyl composition or to the fact that the sodium salts of PA and LPA had been applied as the standards. Based on R_f_s, FA composition, and amounts of FAs, we assume that PA and LPA are represented in areas 2 and 1, respectively. The R_f_ values of lyso-forms of acids are usually lower than that of PA. LPA is characterized by the predominance of saturated FAs and a small amount of phospholipids in a pool. Due to a high content of 16:1Δ9, area 2 probably corresponds to PA [15].

The main FAs in the LPAs of wild-type and transformant cells are represented by 16:0 and 18:0 and then by 16:1Δ9. In the PlsC transformant, the amount of myristic acid reached 12%. The transition from LPA to PA was carried out by LPAAT, which operates at the *sn*-2 position, thus the accumulation of 14:0 occurs at the *sn*-2 position. This suggestion is confirmed by the appearance of 12% of 14:0 in the analyzed area 2 (Table 2).

### 2.3. Analysis of FA Composition in Individual Lipid Classes

Fatty acids analysis of individual glycerolipids from the control showed that all classes contained 16:0 and 16:1 as major FAs. Relatively high levels of myristic acid have been found in all lipid classes. All lipid classes of the transformant cells expressing PlsC revealed a decrease in the amount of 16:0 compared with wild-type cells (Table 3). These changes correlated with the changes in total FA composition (Table 1).

## 3. Discussion

The transition from LPA to PA is carried out by LPAAT, which introduces a particular set of fatty acids at the *sn*-2 position of phospholipids in pro- and eukaryotes. Many bacteria have multiple LPAAT paralogs; these enzymes generate a variety of phospholipids with unique fatty acid compositions and have different FA specificities [15]. The cyanobacterium *Synechococcus elongatus* PCC 7942 has one *plsC* gene for LPAAT, which, according to the FA composition, is specific to 16:0 [8]. Transformation of *Synechococcus* with the gene for LPAAT of the *Cyanobacterium* sp. strain IPPAS B-1200 resulted in the accumulation of 12% of 14:0 at the *sn*-2 position (Table 2). These results correlate with data obtained for the LPAAT of *Cyanothece* sp. PCC 8801, where stereospecific positioning was analyzed with the *sn*-1-specific lipase from *Rhizopus delemar* [3].

Previously, LPAAT from *Cyanothece* sp. PCC 8801 was expressed in *Synechocystis* sp. PCC 6803, resulting in a significant increase in the proportion of myristic acid at the *sn*-2 position in glycerolipids. This result was explained by the high specificity of this enzyme for the 14:0 acyl-carrying protein [3]. In addition, LPAAT from the thermophilic bacterium *Thermus thermophilus* HB8 showed increased specificity for 14:0-CoA and 16:1∆9-CoA substrates [16].

*Synechocystis* sp. PCC 6803 belongs to Group 4 of the cyanobacteria classified according to their FA composition [8]. This strain carries four genes for FADs (namely, *desC* for Δ9-FAD; *desA* for Δ12-FAD; *desD* for Δ6-FAD; and *desB* for ω3-FAD). As a result, it is characterized by a complicated FA composition, consisting of 16:0, 16:1; 18:0, 18:1, 18:2, 18:3, and 18:4 acids, the proportions of which vary depending on growth temperature [17]. Such a model system complicates analysis of the FA composition of the transformants. On the other hand, the primary Δ9-FAD of *Synechocystis* sp. PCC 6803 belongs to type 1 of the Δ9-FADs [8], which desaturate FAs only at the *sn*-1 position [17]. Because 14:0 was assigned to position *sn*-2, its desaturation by the Δ9-FAD of *Synechocystis* was not anticipated [3].

Here, we applied the simple model strain *Synechococcus elongatus* PCC 7942, which belongs to Group 1 and has only one FAD, Δ9-FAD, capable of desaturating the FAs of lipids at the *sn*-1 and *sn*-2 positions [18]. Expression of the *plsC* gene for LPAAT from *Cyanobacterium* sp. IPPAS B-1200 in *S. elongatus* PCC 7942 led to an increase in the proportion of saturated myristic acid up to 26%. However, the latter was not converted into monounsaturated myristoleic acid as expected. This implies that the only Δ9-FAD of *S. elongatus* PCC 7942 is unable to efficiently desaturate 14:0. Expression of the ∆9-FAD gene from *Cyanobacterium* in *S. elongatus* led to an increase in the proportion of monounsaturated acids, particularly in palmitoleic acid, 16:1∆9. Actually, in the absence of C14 FAs, the foreign desaturase duplicated the activity of the native ∆9-FAD of *S. elongatus*. Co-expression of LPAAT and ∆9-FAD of *Cyanobacterium* in *S. elongatus* increased the proportion of 14:1 and 16:1 at the expense of 14:0 and 16:0, suggesting that Δ9-FAD of *Cyanobacterium* is capable of desaturating both saturated FAs, C14 and C16.

The Δ9-FADs of cyanobacteria belong to a class of integral membrane acyl-lipid desaturases that operate on acyl chains attached to the glycerol moiety. The crystal structure of these acyl-lipid desaturases has yet to be determined. However, the structures of functionally comparable stearoyl-CoA desaturases are accessible [19,20]. These enzymes are membrane-integrated homodimers that form a hydrophobic tunnel to accommodate substrates [21]. The tunnel’s length forces carbons 9 and 10 of the acyl chain to align proximal to the relatively buried catalytic metal (Fe or Zn) atoms coupled to His-clusters inside the enzyme’s catalytic site [19]. The shape of the hydrophobic cavity is hypothesized to determine a FAD’s regioselectivity for *cis* dehydrogenation of the substrate [19,20]. The location of a catalytic site in the tunnel may also influence the length of the acyl chain that can be processed by a particular FAD. The latter may explain the observed differences in substrate preference between the ∆9-FADs of *S. elongatus* and *Cyanobacterium*.

Cyanobacteria are distinguished by a wide range of component fatty acids in their lipids, which corresponds to a wide range of phenotypes and environmental conditions, many of which are quite extreme. *S. elongatus* and *Cyanobacterium* sp. both belong to Group I of the cyanobacteria, which possess only ∆9-FAD activity and are capable of synthesizing unsaturated and monounsaturated FAs [8]. This FA composition is characteristic for thermophilic strains that are usually not exposed to low temperatures and cannot synthesize polyunsaturated FAs in response to cold. C14-rich *Cyanobacterium* sp. IPPAS B-1200 was isolated from Lake Balkhash (Kazakhstan) with a salinity of 6.0 g L^−1^. Lake Balkhash is a semi-freshwater lake: the western part of the lake is almost fresh (mineralization is 0.74 g L^−1^), while the eastern part has higher salinity (3.5–6.0 g L^−1^). Water temperature at the surface of the lake varies from 0 °C in December to 28 °C in July. Therefore, *Cyanobacterium* sp. must acclimate to multiple changing parameters, such as temperature, salinity, and maybe pH. It is known that an increase in C14 FAs and 16:0 is a feature of the acclimation to high temperatures [22]. High amounts of C14 FAs may rigidify the membranes, which is important for survival under heat stress.

Myristic acid is a component of cell membranes and seed oils and a source for protein myristoylation [23]. It is applied in various industries and manufacturing processes, e.g., as flow agent and emulsifier in foods and beverages; as a surfactant in soaps, detergents, and textiles; and as a lubricant in plastics. Myristoleic acid is also located in cell membranes and plant oils. Esterified to cetyl alcohol, myristoleic acid is modified to cetyl myristoleate, a drug for osteoarthritis treatment [24].

Myristic and myristoleic acids are usually produced from seeds or Myristicaceae plants, such as nutmeg (*Myristica frangans*). Nutmeg butter is rich in trimyristin (up to 75%)—a triglyceride with three saturated myristic acid residues. Another source is the seeds of African nutmeg (*Pycnanthus angolensis* or *P. kombo*), which contain up to 60% so-called combo oil, rich in myristic (58–64%) and myristoleic (19–26%) acids [25].

Cyanobacteria are fast-growing cells that are cultured in photobioreactors that produce substantial amounts of biomass for various purposes [26]. In particular, *Synechococcus elongatus* PCC 7942 is often used for “light-driven autotrophic cell factories” to produce biofuels and various fine chemicals directly from CO_2_ [27]. The recombinant C14-producing *Synechococcus* strain described here may be used for the biotechnological production of myristic and myristoleic acids.

## 4. Materials and Methods

### 4.1. Cyanobacterial Strains and Growth Conditions

In the course of the experiment, strains of the model cyanobacterium *Synechococcus elongatus* PCC 7942 and the *Cyanobacterium* sp. strain IPPAS B-1200, rich in short FAs, were used. Cultivation was carried out under conditions selected during the isolation of this strain [5]. *Synechococcus* cells were maintained on a solid BG-11 medium [28] with an agar content of 1.2%. The experimental growth conditions were as follows: liquid medium BG-11 with the addition of HEPES-NaOH pH 7.5 in a volume of 250 mL under a constant illumination of 50 µE m^−2^ s^−1^ at 33 °C. The cultures were doused with sterile air that contained 1.5% CO_2_. The samples were fixed in the middle of the exponential growth stage (OD_750_~1); planting and cultivation were done under aseptic conditions.

### 4.2. Cloning and Expression of the desC and plsC Genes

Genomic DNA from *Cyanobacterium* IPPAS B-1200 was isolated according to Williams [29]. The *desC* and *plsC* genes were amplified using primers containing the sequences of certain restriction sites: *desC*1200F ATAACCATGGCAGTTTCAAC; *desC*1200R TTTGAAGCTTTTATTATGCC; *plsC*1200F GAATTAGACCATGGCTAAGG; and *plsC*1200R CTAACACTTGCCTAAGCTTAAAC. Phusion High-Fidelity DNA Polymerase (New England Biolabs, Ipswich, MA, USA) was used for PCR. The amplified DNA fragments were digested with the appropriate restriction enzymes (*Nco* I and *Hin*d III), purified using the Cleanup Standard kit (Evrogen, Ltd., Moscow, Russia), and ligated with the linearized pTrc99A vector (Pharmacea, Uppsala, Sweden; https://www.addgene.org/vector-database/4402 (accessed on 31 December 2023)). The vector was generated in *E. coli* strain XL-1 (Stratagene, La Jolla, CA, USA) and used as a template for further cloning. The genes of interest under the control of the constitutive Trc promoter were amplified into the pAM1303 ([30] https://www.addgene.org/40243 (accessed on 31 December 2023) and pNS2 vectors (the latter vector is similar to pAM1303, but contains other neutral recombination sites (NS2) of the *S. elongatus* genome and a kanamycin resistance cassette). Amplification primers contained the *Xma* I restriction site sequence. The vectors were linearized with this restriction enzyme and treated with FastAP alkaline phosphatase (Thermo Scientific, Waltham, MA, USA). The resulting vectors were used to transform *S. elongatus* PCC 7942 by homologous recombination. The selection of transformants was carried out on a solid BG-11 medium containing spectinomycin and kanamycin at final concentrations of 30 and 25 µg ml^−1^, respectively.

### 4.3. FA Analysis of Total Lipids

Wild-type *S. elongatus* PCC 7942 cells and three transformants (pAM-*desC*, pNS2-*plsC,* and the double transformant pAM-*desC* + pNS2-*plsC*) were used to analyze the FA composition [31]. To 200 µg of harvested cells (wet weight) 1 mL of 80% aqueous ethanol solution of 1 M KOH was added; the samples were vortexed and incubated at 70 °C for 1 h. The samples were then washed twice with 500 µL *n*-hexane to remove unsaponifiable compounds, e.g., free sterols, pigments, etc. The excess KOH was neutralized by adding 50 µL of 20% sulfuric acid to a slightly acidic level (pH of 4.5–4.7 according to the paper test) to the obtained samples. Free FAs were extracted from the samples with *n*-hexane (300 µL) and evaporated to dryness, and 200 µL 1% sulfuric acid in methanol was added. FA methylation was carried out for 30 min at 55 °C. FA methyl esters (FAMEs) were extracted into *n*-hexane in a volume of 200 µL.

### 4.4. GC-MS

FAMEs were analyzed using an Agilent 7890A gas–liquid chromatograph with an Agilent 5975C mass spectrometric detector (Agilent Technology Systems, Santa Clara, CA, USA). A capillary 60 m HP-88 column (inner diameter ∅ 0.25 mm, film thickness 0.2 µm, Agilent J&W, Santa Clara, CA, USA) was used. Other analysis conditions: carrier gas helium, flow rate 1 mL/min, sample volume 1 µL, flow divider 1:20, evaporation temperature 260 °C. Program for gradient analysis: from 130 °C to 170 °C in steps of 6.5 °C/min; 170 to 215 °C in 2.75 °C/min increments; 215 °C for 25 min; 215 to 240 °C in 5 °C/min increments; and a final stage of 50 min at 240 °C. The operating temperature of the mass spectrometric detector was 240 °C; the ionization energy was 70 eV.

### 4.5. Analysis of Lysophosphatidic and Phosphatidic Acids

The total lipid extract was obtained by the classical Bligh-Dyer method [31] and purified through PTFE filters (Agilent, 5190–5265). To isolate lysophosphatidic (LPA) and phosphatidic (PA) acids, the total extract was separated by TLC (Fluka Silica gel TLC Al foils 10 × 10 cm) in hexane:acetone:acetic acid (40:50:2). Phospholipids remained in situ and were detected by staining with primulin (0.05% in acetone:water 80:20); a mixture of 1-oleoyl LPA (http://www.gzsopo.com/product/PNOALD-O353308.html. CAS: 655528-98-5; accessed on 31 December 2023), PA sodium salt (http://www.gzsopo.com/product/PNOMACKLIN-L864045.html. CAS 383907-53-7; accessed on 31 December 2023), and phosphatidylcholine (PC) was used as a standard. The appropriate silica gel sections were taken from the plate and transferred to Eppendorf tubes, where they were washed with a chloroform–methanol (1:1) mixture followed by an equivalent volume of water. The bottom fraction containing phospholipids was chosen after centrifugation. To separate phospholipids, the chloroform:methanol:acetone:water:acetic acid (6:2:8:1:1:1) system was utilized. The LPA and PA standards had R_f_s of 0.4 and 0.7, respectively. To detect lipid groups, primulin staining was applied. Lipids from all four detected groups were isolated, extracted similarly to phospholipids, and treated in methanol with 1% H_2_SO_4_ for 30 min at 55 °C [32]. The FAMEs were then extracted with *n*-hexane and analyzed using GC-MS as described above.

### 4.6. Analysis of Fatty Compositions of Individual Classes of Glycerolipids

The extracts of total lipids were separated by two-dimensional TLC [33] (the solvent for the first dimension was chloroform:methanol:water (75:25:2.5) and for the second was chloroform:methanol:acetic acid:water (80:9:12:2)). The results of separation were visualized using 0.01% primuline in 80% acetone. Each lipid group was extracted and treated in methanol with 1% H_2_SO_4_ for 30 min at 55 °C. The FAMEs were then extracted with hexane and analyzed using GC-MS as described above.

## 5. Conclusions

Our findings reveal that LPAAT from *Cyanobacterium* sp. assures 14:0 synthesis in *S. elongatus* (up to 25%). However, this did not result in any significant 14:1 rise, indicating that *S. elongatus* Δ9-FAD is unable to desaturate FAs shorter than 16:0. Co-expression of LPAAT and Δ9-FAD (*desC*) of *Cyanobacterium* sp. in *S. elongatus* resulted in the accumulation of 14:0, 14:1, and 16:1 in *Synechococcus* cells, indicating that Δ9-FAD of *Cyanobacterium* has a broad range of length specificity and can accommodate and desaturate both C14- and C16-saturated FAs. Thus, the acyltransferase LPAAT, which drives 14:0 synthesis, and Δ9-FAD, which can accommodate and dehydrogenate myristic acid as a substrate, ensure unusual C14-rich (up to 40%) FA composition in *Cyanobacterium* sp. IPPAS B-1200. Recombinant C14-producing *Synechococcus* cells may be used for biotechnological production of myristic and myristoleic acids.

## Figures and Tables

**Table 1:** FA composition (mass %) of total lipids of *Synechococcus elongatus* PCC 7942 wild-type (7942) and its transformants expressing LPAAT *plsC1200* (*plsC*) and Δ9-FAD *desC1200* (*desC*) and co-expressing *plsC1200* together with *desC1200* (*plsC* + *desC*).

FAs	7942	*plsC*	*desC*	*plsC + desC*
14:0	0.5	10.2	0.6	26.5
14:1∆9	0.5	0.8	9.0	9.0
16:0	50.3	40.4	16.0	15.2
16:1∆9	31.7	34.6	73.1	48.5
18:0	1.2	2.6	0.6	0.2
18:1∆9	13.0	9.4	0.5	0.5
18:1∆11	2.8	2.0	0.2	0.1

FAs: 14:0—myristic acid; 14:1∆9—myristoleic acid; 16:0—palmitic acid; 16:1∆9—palmitoleic acid; 18:0—stearic acid; 18:1∆9—oleic acid; 18:1∆11—*cis*-vaccenic acid. Individual peaks were identified with Agilent MSD ChemStation 4.0.3 software and the NIST library. The experiments have been repeated three times. Standard deviations are in the range of 0.1–0.3%.

**Table 2:** FA compositions of LPA and PA (mass %) obtained by separation of phospholipid fractions (Figure 2). WT, phospholipids from *S. elongatus* PCC 7942 wild-type cells; PlsC, phospholipids from *S. elongatus* PCC 7942 cells expressing PlsC (LPAAT) from *Cyanobacterium* sp. IPPAS 1200.

	WT		PlsC	
FAs	LPA	PA	LPA	PA
14:0	0.8	0.6	3	12.3
14:1Δ9	*	1	0.5	1.2
16:0	57.4	49.4	53.6	38
16:1Δ9	4	32.3	4.8	35.2
18:0	37.1	9	37.5	8
18:1Δ9	0.7	7.7	0.6	5.3

* At standard parameters (width: 2–5 s, threshold: 5, base in fractions of width: 0.5 Min, mV: 0) the peak was not integrated, but the spectrum of the corresponding methyl esters could be detected. Standard deviations are in the range of 0.1–0.3%.

**Table 3:** The content of FAs in individual areas (mass %) obtained by separation of glycerolipid fractions. WT, glycerolipids from *S. elongatus* PCC 7942 wild-type cells; PlsC, glycerolipids from *S. elongatus* PCC 7942 cells expressing PlsC (LPAAT) from *Cyanobacterium* sp. IPPAS 1200. MGDG—monogalactosyl diacylglycerol; DGDG—digalactosyl diacylglycerol; SQDG—sulfoquinovosyl diacylglycerol; PG—phosphatidylglycerol.

	WT				PlsC			
FAs	MGDG	DGDG	SQDG	PG	MGDG	DGDG	SQDG	PG
14:0	1.2	2.3	1.1	0.9	14.6	19.7	7.6	7.9
14:1Δ9	0.1	1.1	0.8	1.0	1.2	0.8	0.6	1.7
16:0	48.1	40.3	56.2	49.5	31.2	30.9	48.7	41.9
16:1Δ9	46.2	42.9	34.3	32.3	48.1	43.4	35.5	33.0
18:0	0.8	10.7	6.0	5.2	1.1	2.1	3.2	4.7
18:1Δ9	3.6	2.7	1.6	11.1	3.8	3.1	4.4	10.8

## Data Availability

Data are contained within the article.
